# A PRISMA Systematic Review of Sexual Dysfunction and Probiotics with Pathophysiological Mechanisms

**DOI:** 10.3390/biology14030286

**Published:** 2025-03-11

**Authors:** Su-Jin Yang, Trang Thi Minh Nguyen, Xiangji Jin, Qiwen Zheng, Se-Jig Park, Gyeong-Seon Yi, Tae-Hoo Yi

**Affiliations:** 1 Graduate School of Biotechnology, Kyung Hee University, 1732 Deogyeong-daero, Giheung-gu, Yongin-si 17104, Republic of Korea; stella@khu.ac.kr (S.-J.Y.); trangnguyen@khu.ac.kr (T.T.M.N.); zhengqiwen@khu.ac.kr (Q.Z.); tpwlt@khu.ac.kr (S.-J.P.); 2 Department of Dermatology, School of Medicine, Graduate School, Kyung Hee University, 26 Kyungheedae-ro, Dong-daemun, Seoul 02447, Republic of Korea; hyanghe112@khu.ac.kr; 3 Department of Biopharmaceutical Biotechnology, Graduate School, Kyung Hee University, 1732 Deogyeong-daero, Giheung-gu, Yongin-si 17104, Republic of Korea; ks010924@khu.ac.kr

**Keywords:** sexual dysfunction, pathophysiological mechanisms, probiotics, PRISMA, systematic review

## Abstract

Sexual dysfunction, influenced by hormonal imbalances, psychological factors, and chronic diseases, affects a significant portion of the population. Probiotics, known for their beneficial effects on gut microbiota, have emerged as potential therapeutic agents for improving sexual health. This systematic review evaluates the impact of probiotics on sexual function, hormonal regulation, and reproductive outcomes. A comprehensive search identified 3308 studies, with 12 meeting the inclusion criteria—comprising 10 randomized controlled trials (RCTs) and 2 in vivo and in vitro studies. Probiotic interventions were shown to significantly improve sexual function, particularly in women undergoing antidepressant therapy (*p* < 0.05). Significant improvements in Female Sexual Function Index (FSFI) scores were observed, with combined treatments such as Lactofem with Letrozole and Lactofem with selective serotonin reuptake inhibitors (SSRIs) demonstrating a 10% biochemical and clinical pregnancy rate compared to 0% in the control group (*p* = 0.05). Probiotic use was also associated with a 66% reduction in menopausal symptoms, increased sperm motility (36.08%), viability (46.79%), and morphology (36.47%). Probiotics also contributed to favorable hormonal changes, including a reduced luteinizing hormone (LH) to follicle-stimulating hormone (FSH) ratio (from 3.0 to 2.5, *p* < 0.05) and increased testosterone levels. Regarding reproductive outcomes, probiotic use was associated with higher pregnancy rates in women undergoing fertility treatments and improvements in sperm motility, viability, and morphology in men. This review highlights the promising role of probiotics in addressing sexual dysfunction and reproductive health, suggesting their potential as adjunctive treatments for conditions such as depression and infertility. Further research is needed to better understand the underlying mechanisms of these beneficial effects.

## Simple Summary

Sexual dysfunction, which can result from hormonal imbalances, stress, and chronic health issues, affects a significant portion of the population. This study examines how probiotics, beneficial bacteria that support gut health, can improve sexual and reproductive health. The findings show that probiotics significantly improved sexual function in women, particularly those on antidepressants, and increased pregnancy rates in women undergoing fertility treatments. In men, probiotics improved sperm health, including motility and viability. Additionally, probiotics help reduce menopause symptoms and support hormonal balance. This review highlights the potential of probiotics as an effective treatment for sexual dysfunction and reproductive health, offering promising results that could benefit many individuals. However, further research is needed to fully understand the mechanisms behind these effects.

## 1. Introduction

Sexual dysfunction, affecting approximately 43% of women and 31% of men in the United States, profoundly impacts quality of life [1]. This issue is commonly associated with hormonal imbalances, chronic conditions such as diabetes and hypertension, and psychological factors [2]. The DSM-5 identifies conditions like female sexual interest/arousal disorder and genito-pelvic pain/penetration disorder, with symptoms persisting for at least six months and causing significant distress [3]. Among cancer patients, sexual dysfunction is prevalent, with treatments linked to a roughly three-fold increase in risk for both cervical and breast cancer [2]. Despite its widespread occurrence, sexual dysfunction often goes undiagnosed due to stigma and insufficient clinical training. Diagnostic tools such as the Female Sexual Function Index (FSFI) are instrumental in assessing sexual health [4]. For women, evidence-based treatments include hormone therapies, such as transdermal testosterone, and pelvic floor physical therapy, particularly for hypoactive sexual desire disorder and dyspareunia [3]. Psychological interventions, including mindfulness and cognitive–behavioral therapy, also contribute to effective management [1]. In men, erectile dysfunction is frequently associated with vascular or neurological causes, with first-line treatments like lifestyle modifications and phosphodiesterase type 5 inhibitors demonstrating significant efficacy [5]. The complexity of sexual dysfunction, especially in the context of cancer [2], highlights the critical need for continued research to enhance diagnostic accuracy, optimize treatment strategies, and improve patient outcomes.

Pathophysiological mechanisms involved in sexual dysfunction are closely linked to the gut microbiota, a crucial regulator of metabolism, immunity, and overall health [6,7,8,9]. Dysbiosis, or imbalance in the gut microbiota, is associated with metabolic disorders, including type 2 diabetes [10]. The gut microbiota produces metabolites such as short-chain fatty acids (SCFAs) that interact with the nervous, immune, and metabolic systems, impacting systemic health [11]. Recent research has identified the gut–brain axis as a key pathway through which gut microbiota influences sexual function by regulating neural signaling and hormone metabolism [12]. Specifically, the gut microbiota plays a critical role in modulating sex hormones such as estrogen and testosterone, which are essential for maintaining sexual health [8,13,14]. In diabetic individuals, dysbiosis exacerbates sexual dysfunction through mechanisms including increased inflammation, oxidative stress, and impaired vascular function, all of which are influenced by the gut microbiota [8,15]. Restoring a balanced microbiota may provide promising therapeutic strategies for improving sexual health in patients with diabetes [16].

Probiotics are emerging as a potential solution for sexual dysfunction, especially in patients experiencing medication-induced sexual health issues, such as those caused by selective serotonin reuptake inhibitors (SSRIs). Research has shown that probiotics, including strains like *Lactobacillus acidophilus* and *Bifidobacterium bifidus*, not only promote gut microbiome balance but also impact the neuroendocrine systems associated with sexual function. A randomized trial by Hashemi-Mohammadabad et al. (2023) demonstrated that probiotic supplementation improved sexual satisfaction and alleviated depressive symptoms in SSRI-treated patients, suggesting potential beyond gut restoration [17]. Probiotics may exert their beneficial effects through mechanisms such as reduced systemic inflammation, enhanced serotonin production in the gut, and improved hormonal regulation—all of which contribute to sexual health [18]. The gut–brain axis regulates serotonin production, alleviating depression [19,20], a major cause of sexual dysfunction [21,22]. Probiotics modulate key sex hormones like estrogen and testosterone [22,23] and possess antioxidant properties that combat oxidative stress, protecting tissues [24] involved in sexual function. Given that the American Urological Association (AUA) and the International Society for Sexual Medicine (ISSM) have highlighted the role of gut health in sexual function, probiotics are becoming recognized as a promising adjunctive therapy for sexual dysfunction [25,26]. The growing evidence points to the need for more clinical trials and guideline-based recommendations to incorporate probiotics as a therapeutic option, particularly for those affected by drug-induced sexual health disturbances.

The objective of this study is to systematically examine the potential role of probiotics as a therapeutic intervention for diabetes-related sexual dysfunction. Specifically, the review focuses on understanding how probiotics can modulate key mechanisms such as hormonal regulation and metabolic pathways. By synthesizing findings from in vitro, in vivo, and clinical studies, the research highlights the role of gut microbiota in influencing sexual health and identifies probiotics as a potential adjunct therapy. The study also aims to address knowledge gaps regarding strain-specific effects and long-term safety, paving the way for future research and clinical applications.

## 2. Materials and Methods

This systematic review was conducted following the Preferred Reporting Items for Systematic Reviews and Meta-Analyses (PRISMA) 2020 guidelines to explore the potential therapeutic role of probiotics in managing sexual dysfunction and its associated pathophysiological mechanisms. The primary objectives were to address the following research questions:

1. What evidence exists from in vitro, in vivo, and clinical studies on the effects of probiotics on sexual dysfunction?
2. How do probiotics influence key pathophysiological mechanisms underlying sexual dysfunction, including inflammation, oxidative stress, and hormonal imbalances?

A comprehensive literature search was conducted across multiple electronic databases, including PubMed, Scopus, and Web of Science. The search included all publications available up to August 2024. Search terms included combinations of keywords “probiotics” and “sex” or “sexual function”. Specific terms related to sexual function in MESH terms included “Sexual Dysfunction, Physiological”, “Dyspareunia”, “Ejaculatory Dysfunction”, “Premature Ejaculation”, “Retrograde Ejaculation”, “Erectile Dysfunction”, “Impotence, Vasculogenic” and “Vaginismus”.

### 2.1. Inclusion and Exclusion Criteria

Studies were included if they investigated the effects of probiotics on sexual dysfunction, were published in peer-reviewed journals, written in English, and conducted as experimental studies (in vivo, in vitro) or epidemiological studies, including clinical trials. Studies lacking original experimental or clinical data, including review articles, meta-analyses, guidelines, protocols, case series, case reports, and conference abstracts, were excluded. Research investigating non-probiotic interventions, such as pharmaceutical agents, herbal extracts, or dietary modifications without a probiotic component, was not considered. Exclusion also applied to studies combining probiotics with other therapeutic modalities without isolating their specific effects. Preclinical animal studies focusing on unrelated conditions and publications in languages other than English or with inaccessible full texts were omitted.

### 2.2. Study Selection Process

Two independent reviewers, T.T.M.N. and S.J.Y., independently screened the titles and abstracts of identified studies to determine their relevance to the topic of probiotics on sexual function. Each full-text article was systematically evaluated based on the predefined inclusion and exclusion criteria to confirm its eligibility. Any reviewer inconsistencies were addressed through discussion to maintain consistency and reduce selection bias. In cases where consensus could not be reached, a third reviewer was consulted to provide a final determination.

### 2.3. Data Extraction and Synthesis

Data were extracted from the included studies, focusing on three primary areas. First, sexual function outcomes were assessed using validated tools such as the FSFI and other relevant measures. Second, hormonal markers were analyzed, including changes in hormone levels (e.g., estrogen, testosterone, LH/FSH ratio). Third, reproductive outcomes were evaluated by examining pregnancy rates, sperm parameters, and menopausal symptom relief. Data extraction included clinical assessments, biochemical analyses, and microbiome evaluations, with an emphasis on strain-specific effects. The synthesis aimed to provide a comprehensive understanding of the mechanisms by which probiotics influence sexual function, hormonal balance, and reproductive health.

## 3. Results

**Figure 1:**
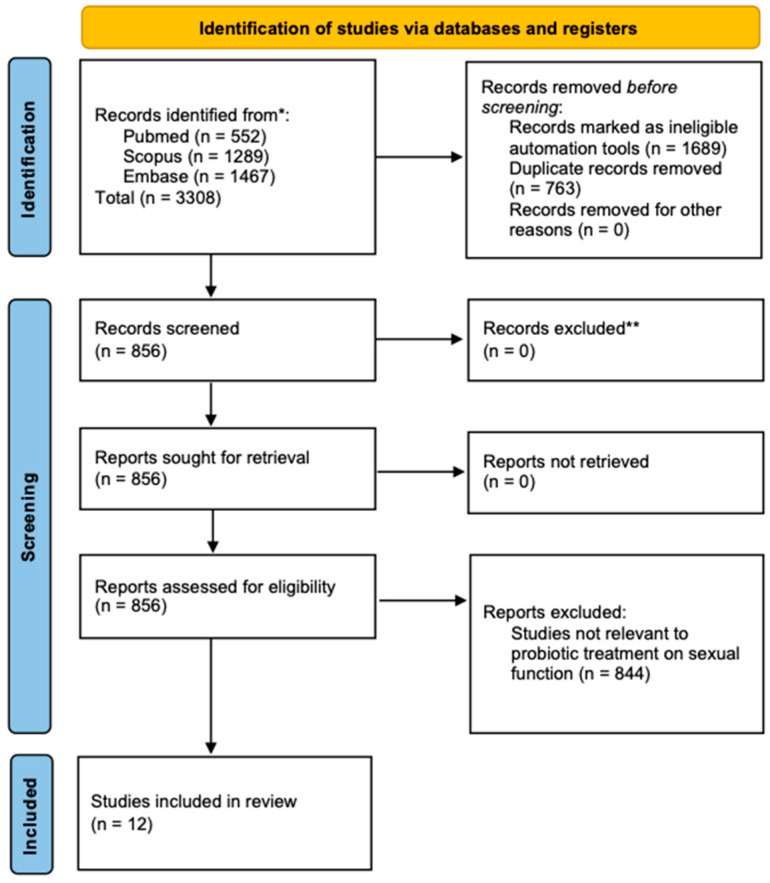
A diagram outlining the systematic review process, including database searches, abstract screenings, and full-text acquisitions, in compliance with PRISMA guidelines. * Data collected in August 2024; ** Studies with incomplete data were excluded.

A total of 3308 studies were identified through the initial search (Figure 1) following the PRISMA table (Supplement File S1). After applying inclusion and exclusion criteria, 12 studies were included in the final synthesis on specific parameters (Table 1). The most frequently studied strain was *Lactobacillus acidophilus* (*L. acidophilus*), with Iran being the leading contributor to these studies (Table 2). These studies varied in methodology, including 10 randomized controlled trials (RCTs) and two in vivo and in vitro studies exploring the effects of probiotics on sexual dysfunction through (1) improvements in sexual function scores, (2) impacts on hormonal markers, and (3) pregnancy and reproductive outcomes.

### 3.1. Improvement in Sexual Function Scores

Several studies in the reviewed literature demonstrated significant improvements in sexual function scores following probiotic interventions. Kutenaee et al. [27] and Hashemi-Mohammadabad et al. [17] both reported improvements in the FSFI scores, with Kutenaee et al. noting a significant enhancement in the Lactofem plus Letrozole group compared to Letrozole alone (*p* < 0.05). Similarly, Hashemi-Mohammadabad et al. found that the Lactofem plus SSRIs group showed significant improvements in FSFI domains and total scores compared to SSRIs alone (*p* < 0.05). Hashemi et al. (Iran) further supported these findings, reporting that the Lactofem group showed better sexual desire, arousal, lubrication, orgasm, satisfaction, and pain dimensions compared to the SSRIs-only group (*p* < 0.05) [17]. Lim et al. [31] conducted an RCT in Korea with 85 post-menopausal women, evaluating the effects of *Lactobacillus acidophilus* (*L. acidophilus*) YT1, showing a 66% reduction in menopausal symptoms, compared to 37% in the placebo group. *L. acidophilus* YT1 alleviated symptoms such as hot flashes, fatigue, and vaginal dryness, without changes in estrogen levels, suggesting it may improve sexual function by regulating the gut microbiome, immune system, and central nervous system. These findings collectively suggest that probiotics, either alone or in combination with other treatments, can significantly enhance sexual function in women, particularly those with conditions like those undergoing antidepressant therapy.

### 3.2. Impact on Hormonal Markers

Probiotic interventions were also associated with positive changes in hormonal and inflammatory markers, which may contribute to improved sexual health. Kutenaee [27] reported a significant decrease in the luteinizing hormone (LH) and follicle-stimulating hormone (FSH) ratio in the probiotics group (from 3.0 to 2.5, *p* < 0.05), indicating improved hormonal balance. Hashemi et al. [17] also noted a significant reduction in depressive symptoms, which are often linked to hormonal imbalances, in the Lactofem group compared to the SSRIs-only group (*p* < 0.05). Increased serum markers included elevated total antioxidant capacity (TAC), LH, FSH, and testosterone levels (*p* < 0.05), as reported by Ansari et al. [37]. These findings indicate that probiotics may improve sexual function by modulating hormonal and inflammatory pathways, particularly in individuals with conditions like depression and diabetes.

### 3.3. Pregnancy and Reproductive Outcomes

Probiotic interventions demonstrated significant improvements in reproductive outcomes. Kutenaee et al. [27] reported higher biochemical and clinical pregnancy rates in the probiotics plus Letrozole group (10%) compared to the Letrozole-alone group (0%) (*p* = 0.05). Hashemi et al. [17] found that 8 weeks of probiotic consumption improved chemical and clinical pregnancy rates. In male reproductive health, Ansari et al. [37] reported that *B. longum* and *Cynara scolymus* L. extract increased sperm motility (36.08%), viability (46.79%), and morphology (36.47%) in diabetic male rats. Similarly, Abbasi et al. [36] showed that the synbiotic product FamiLact significantly improved sperm concentration (44.73 ± 10.02 vs. 23.27 ± 5.19 million/mL), motility (42.2 ± 5.63% vs. 19.4 ± 4.24%), and morphology (48.6 ± 8.56% vs. 25.8 ± 7.05%) while reducing DNA fragmentation (*p* < 0.05) in men with idiopathic infertility. These findings indicate that probiotics contribute to enhanced pregnancy outcomes, sperm quality, and overall reproductive health, particularly in individuals with underlying reproductive issues.

## 4. Discussion

This systematic review integrates findings from 12 studies encompassing randomized controlled trials, in vivo experiments, and in vitro analyses to assess the impact of probiotics on sexual dysfunction. The aggregated evidence indicates that probiotics may substantially enhance sexual function scores, regulate hormonal profiles, and improve reproductive outcomes. These results underscore the multifaceted role of probiotics in modulating physiological and psychological factors linked to sexual health, offering promising insights into their therapeutic potential.

### 4.1. Probiotics and Sexual Function Enhancement

The reviewed studies highlight that probiotics can improve sexual function, especially in individuals experiencing dysfunction due to antidepressant treatment or menopausal symptoms. Probiotic interventions, such as Lactofem in combination with Letrozole or selective serotonin reuptake inhibitors (SSRIs), have shown significant improvements in FSFI scores, with enhanced sexual function and reduced symptoms such as vaginal dryness and fatigue [17,27,31]. The underlying mechanisms appear to be multifactorial, involving modulation of the gut–brain axis [38], regulation of immune responses, and neurochemical pathways that impact mood and sexual health [39,40]. Neurotransmitters such as serotonin, dopamine, gamma-aminobutyric acid, and glutamate [41,42] play vital roles in the connection between the gut and brain, influencing both mental and physical processes [38]. Unlike traditional antidepressants, probiotics do not seem to alter sensitivity to positive or negative emotions [43]. Additionally, probiotics have been found to enhance cognitive adaptability, reduce stress in older adults, and bring about beneficial changes in gut microbial composition [42]. For instance, *L. acidophilus* YT1 has shown effectiveness in reducing menopausal symptoms without altering estrogen levels, indicating that gut microbiota modulation may work through more indirect pathways [31].

In comparison to conventional interventions such as SSRIs or hormone replacement therapy (HRT), probiotics offer a more natural and integrative alternative. SSRIs are effective in the treatment of depression, but they often induce sexual side effects, including reduced libido and delayed orgasm [44]. While HRT can ameliorate sexual dysfunction in menopausal women, it is frequently associated with long-term health risks [45,46]. In contrast, probiotics provide a promising adjunctive treatment with minimal adverse effects, supporting sexual health through modulation of the gut microbiota, immune regulation, and neurochemical signaling [47,48,49,50]. Emerging research underscores the potential of probiotics, like *Lactobacillus plantarum* 299v, to enhance cognitive performance, reduce systemic inflammation, and improve sexual well-being, presenting a valuable and safer complementary strategy to traditional pharmacological approaches [47,48,49,50].

### 4.2. Hormonal Modulation Through Probiotic Use

Probiotics offer a distinctive and natural approach to hormonal regulation, contrasting favorably with conventional treatments [51,52,53]. While HRT remains the standard for managing sex steroid deficiencies in postmenopausal women, it comes with notable risks, such as cardiovascular complications and breast cancer, with prolonged use [54,55]. Studies have demonstrated that probiotics, such as *Lactobacillus rhamnosus* GG and *Escherichia coli Nissle* 1917, modulate the gut microbiome and immune responses, reducing systemic inflammation and improving levels of hormones like LH, FSH, and testosterone [56,57]. Moreover, probiotics address sex steroid deficiency-related issues [56], such as bone loss and metabolic dysfunction, through mechanisms that involve reducing gut permeability and inflammatory cytokines [58,59,60,61], showcasing their multifaceted role in supporting hormonal health. Probiotics support hormonal health by reducing gut permeability, which prevents the translocation of inflammatory cytokines that can disrupt endocrine function [62,63]. This positions probiotics as a promising adjunctive treatment for hormonal regulation, offering a safer, non-pharmacological alternative to HRT and SSRIs.

### 4.3. Influence on Fertility and Reproductive Health

Probiotics have shown considerable promise in enhancing fertility and reproductive health outcomes [64,65] by modulating the gut microbiota and reducing oxidative stress [66,67,68]. Clinical studies report improved pregnancy rates and sperm parameters when probiotics are combined with conventional treatments [17,27,36,37]. Supplementation with specific probiotic strains has been associated with increased sperm concentration, motility, and morphology, along with reduced DNA fragmentation in men with idiopathic infertility [36]. By restoring gut microbial balance, probiotics help reduce inflammatory cytokines and oxidative markers that negatively impact reproductive function [69]. Unlike antioxidant supplements, which primarily target oxidative stress, probiotics provide comprehensive immune and metabolic regulation [70]. Hormonal therapies, while effective, may have side effects and do not address the systemic imbalances that probiotics can correct [71,72]. Probiotics thus present a multifaceted, non-pharmacological strategy for improving reproductive health, offering distinct advantages over traditional treatments by addressing root causes through gut microbiota modulation and systemic health enhancement [73,74].

### 4.4. Limitations

While the results are promising, several limitations must be acknowledged. The included studies varied in sample size, probiotic strains, dosages, and treatment durations, which may affect the generalizability of the findings. Heterogeneity in probiotic strains and dosages across studies complicates the comparison of results and makes it difficult to determine the most effective probiotic for sexual function management. Additionally, most studies focused on female populations, with limited research on male populations, making it challenging to assess whether the observed benefits are applicable across sexes. The variable quality of the included studies, particularly concerning their experimental design and controls, limits the reliability of the conclusions drawn. Lastly, there is limited long-term follow-up data, which means the sustainability of any observed effects on sexual function is uncertain.

## 5. Conclusions

Probiotic interventions have demonstrated promising potential in improving sexual function, modulating hormonal markers, and enhancing reproductive outcomes. These findings underscore the therapeutic value of probiotics as a complementary treatment for sexual dysfunction, particularly among individuals with underlying health conditions such as depression, infertility, and hormonal imbalances. The studies included in this review highlight significant improvements in sexual function, hormonal regulation, and reproductive health following probiotic interventions. While the results indicate that probiotics can be an effective adjunct therapy for improving sexual function and reproductive health, further research is necessary to establish standardized treatment protocols and explore the long-term impact of probiotics on sexual health.

- Probiotics enhance sexual function and satisfaction in Female Sexual Function Index scores.
- Probiotics improve hormonal balance, lowering LH/FSH and increasing testosterone.
- Probiotics enhance reproductive outcomes with respect to pregnancy rates and sperm quality.
- Probiotics are a promising adjunct for sexual dysfunction treatment.
- Future studies are needed to standardize protocols and explore long-term impacts.

Integrating probiotics as part of a multifaceted management approach could provide patients with a non-pharmacological, cost-effective therapeutic option to address sexual dysfunction, hypoandrogenism, and reproductive dysregulation, thereby enhancing overall health-related quality of life.

## Figures and Tables

**Table 1:** Key parameters of sexual and reproductive health influenced by probiotics.

Category	Parameters Influenced
1. Neurological and functional improvements	↑ FSFI scores
	↓ Vaginal dryness and menopausal symptoms
	↑ Serotonin and dopamine signaling
	↓ Depressive symptoms
2. Hormonal and endocrine modulation	↑ Testosterone levels
	Improved LH/FSH balance
3. Reproductive and fertility outcomes category	↑ Sperm motility, viability, and morphology
	↑ Pregnancy rates

The upward arrow (↑) represents an increase, whereas the downward arrow (↓) represents a decrease.

**Table 2:** Overview of the characteristics of the studies included in the systematic review.

Author Study Type Country	Sample Size	Age	Study Design	Outcome Measures	Follow-Up	Finding
Kutenaee RCT Iran [27]	G-A (LactoFem + Retrozole, *n* = 20) G-B (Retrozole, *n* = 20)	Married woman: 18–38 y/o	G-A: one Lactofem capsule (including *L. acidophilus, B. bifidus, L. rutri*, and *L. fermentum* 2 × 10^9^; capsule weight of 500 mg per bio-capsule) G-B: Letrozole alone.	Demographic data; pregnancy: biochemical pregnancy (β-hCG), clinical pregnancy (ultrasound); ovular follicles and endometrial thickness; menstrual history; hair loss; global acne rating scale; sexual function FSFI; body image; biochemical assessment: total testosterone (ng/mL) and LH/FSH concentrations	2 months	FSFI: improvement in sexual function (significant enhancement in the Lactofem plus Letrozole group). BICI: decreased dissatisfaction with body image. US: changes in follicle size and endometrial thickness observed. Biochemical pregnancy rate: 10% (probiotics plus Letrozole group) vs. 0% (Letrozole alone group), *p* = 0.05. Clinical pregnancy rate: 10% (probiotics plus Letrozole group) vs. 0% (Letrozole alone group), *p* = 0.05. Body image score: improvement in the probiotics G-(30 (4.99) vs 40 (4.36); *p* < 0.01).
Hashemi RCT Iran [17]	G-A: SSRI antidepressants and Lactofem (*n* = 58) G-B: SSRI antidepressants (*n* = 54)	18–45 y/o	G-A: SSRI antidepressants and Lactofem (500 mg bio-capsule containing *L. acidophilus*, *B. bifidum*, *L. rutri*, and *L. fermentum*, 2 × 10^9^ cfu/g) taken orally every day. G-B: SSRI antidepressants only.	Demographic data Sexual function FSFI Sexual satisfaction questionnaire Depressive severity Hamilton Depression Rating Scale Side effects: recorded symptoms included fever, itching, diarrhea, vomiting, or other gastrointestinal issues	2 months	After 8 weeks, the Lactofem plus SSRIs G-showed significant improvements in FSFI domains and total scores compared to the SSRIs alone group (*p* < 0.05).
Wallace RCT Canada [28,29]	*n* = 10	25.2 ± 7 y/o	Probio’Stick^®^ group: 6 × 10^9^ CFU daily. Placebo group: placebo sachet daily.	Depressive symptoms (MADRS); anxiety, sexual function, sleep quality, neuroimaging, and molecular analysis; polysomnography for sleep quality; molecular analysis: blood, urine, and stool samples for DNA, RNA, and microRNA sequencing; neuroimaging: MRI scan	Duration: 16 weeks in total (8 weeks divided into two stages)	Depression (MADRS): 24.9 ± 3.4 ⟶ 15.4 ± 4.4 (week 4, *p* < 0.001) ⟶ 12.7 ± 8.1 (*p* = 0.377). Depression (QIDS-SR16): 20.5 ± 6.4 ⟶ 14 ± 6.3 (week 4, *p* < 0.001) ⟶ 11.6 ± 8.1 (*p* = 0.126). Anhedonia (SHAPS): 36.8 ± 2 ⟶ 31.4 ± 4.6 (week 4, *p* = 0.004) ⟶ 30.7 ± 6.8 (*p* = 1.000). Anxiety (GAD-7): 13.5 ± 3.8 ⟶ 9.6 ± 4.9 (week 4, *p* = 0.021) ⟶ 9.8 ± 6.6 (*p* = 1.000). Anxiety (STAI): 123.3 ± 11.3 ⟶ 105.7 ± 21.5 (week 4, *p* = 0.016) ⟶ 104.3 ± 28.1 (*p* = 1.000). Sleep quality (PSQI): 9.8 ± 3.6 ⟶ 8.6 ± 2.9 (*p* = 0.167) ⟶ 6.6 ± 2.8 (week 8, *p* = 0.002). Safety: no side effects or adverse events.
Xiao RCT China [30]	*n* = 12	Metabolic syndrome (MetS) between 30 and 65 y/o	Probiotic group: two sachets of 1:1 mixture of *B. adolescentis* CCFM8630 and *L. reuteri* CCFM8631 (1 × 10^10^ CFU) daily. Placebo: maltodextrin.	BMI and waist circumference; hip circumference, waist-to-hip ratio, fasting blood glucose, fasting insulin, HbA1c, CRP, total cholesterol (TC), triglycerides (TG), HDL, and LDL; fecal analysis GC-MS and 16S rRNA gene amplicon sequencing	11 weeks	*B. adolescentis* CCFM8630 and *L. reuteri* CCFM8631 show promising effects as an adjuvant therapy for metabolic syndrome, significantly reducing fasting blood glucose, fasting insulin, triglycerides, and LDL-C. The intervention also increased the proportion of bacteroides and levels of acetic acid, propionic acid, and butyric acid, as well as the abundance of phascolarctobacterium and lachnospira.
Lim RCT Korea [31]	*n* = 85 (42 experimental, 43 control)	45 and older with postmenopausal symptoms	Probiotic group: *L. acidophilus* YT1 MENOLACTO^®^ (1 × 10⁸ CFU/day). Placebo group: an identical placebo.	MENQOL (Menopause-Related Quality of Life); KMI (Kupperman Menopausal Index): Evaluates menopausal symptoms such as hot flashes, insomnia, nervousness, and fatigue	12 weeks with visits every 6 weeks	MENOLACTO^®^ resulted in a 66% reduction in menopausal symptoms, compared to a 37% reduction in the placebo group. Unlike hormone replacement therapy (HRT), which alleviates symptoms but carries risks such as heart disease and stroke, MENOLACTO^®^ improved symptoms without these risks. Phytoestrogens, while evaluated, have shown mixed results. Probiotics like *L. acidophilus* regulate the gut microbiome, immune system, and central nervous system, effectively alleviating symptoms such as hot flashes, fatigue, and vaginal dryness, regardless of estrogen and FSH levels.
Rapisarda RCT Italy [32]	*n* = 60 Active group *n* = 40 Placebo group *n* = 20	32.93 ± 7.58 y/o	Active group: *L. rhamnosus* CA15 (DSM 33960) oral capsules 10 1^0^ log CFU/g once daily. Placebo group: received the same capsule with corn starch.	Clinical signs and symptoms: severity score; vaginal fluid pH; nugent score; Lactobacillary Grade (LBG)	T0: 10 days T1: 30 days after end of treatment (washing, T2)	Impact of *L. rhamnosus* CA15: significantly inhibits sperm activity and reduces pregnancy rates through its adhesion properties.
Orazov RCT USA [33]	Postmenopausal atrophic vaginitis patients *n* = 69 G-1 *n* = 34 G-2 *n* = 35	53.6 ± 2.1 y/o)	G-1: estriol monotherapy, 0.5 mg intranasally, administered daily. G-2: treatment: intra-vascular combination therapy with 0.2 mg estriol, 2.0 mg of micronized progesterone *L. casei* rhamnosus dodderlane (LCR), and 341 mg lyophilized culture.	Nappi RE Scale; D. Barlow Scale; vaginal pH measurement; colpocytology; Bachmann’s Vaginal Health Index; colposcopy; sonography; pipelle biopsy (if needed); pap test	Treatment period: 12 weeks Follow-up period: 12 weeks after treatment	G2: Significant improvement in vulva/vaginal mucosa (pale pink, sufficient moisture) and symptoms (itching, burning, dryness, dyspareunia). Dyspareunia relief: G1: 52.9% relief at 12 weeks and 70.6% complete relief at 24 weeks. G2: 71.5% relief at 12 weeks and 97.1% complete relief at 24 weeks (*p* < 0.05). Objective symptoms: G2 showed greater improvements in elasticity (77.1% vs 55.9%) and epithelial thickness (85.7% vs 76.5%) at 12 weeks (*p* < 0.05). At 24 weeks: continued improvement in G2 (elasticity at 85.7% and epithelial thickness at 77.1%). Other parameters (no sig. diff.): no changes in vaginal folds, fluid secretion, moisture, color, D. Barlow Scale, pH, or Bachmann’s Index. pH: 4.1 ± 0.3; Bachmann’s Index: 4.65 ± 0.59 after 3 months. Colposcopy: No abnormal findings post-treatment.
Khodaverdi RCT Iran [34]	*n* = 37 Probiotic group (LactoFem^®^): 18 Placebo group: 19	18–45 y/o women with stage III-IV endometriosis and VAS Pain Score: initial score > 4	Treatment group: LactoFem^®^ probiotic capsule containing *L. acidophilus*, *L. plantarum*, *L. fermentum*, and *L. gasseri*; 1 capsule daily. Control group: identical placebo capsules.	Dysmenorrhea; chronic pelvic pain; overall pain scores; safety	12 weeks	Dysmenorrhea: significant pain reduction in the first 8 weeks (*p* = 0.018); no change after (*p* > 0.05). Chronic pelvic pain: greater reduction with probiotics (week 0–8, *p* = 0.119), but placebo improved more (week 8–12, *p* = 0.02). Overall pain: significant reduction with probiotics (week 0–8, *p* = 0.017); continued difference (week 8–12, *p* = 0.015).
Mollazadeh-Narestan RCT Iran [35]	*n* = 18 women with VVC (*Candida* spp.)	15–49 y/o	Fluconazole group: one red capsule containing 150 mg fluconazole. Placebo group: 30 white capsules of placebo (potato starch). Probiotic group: 30 white capsules of probiotic (*L. acidophilus* LA-5, 1 × 10^9^ CFU/g).	Vaginal pH; microbiological tests; clinical symptoms (vaginal discharge, itching, burning, inflammation, erythema, dysuria, and dyspareunia); patient satisfaction and side effects.	Initial Reminder: days 12–15 First FU: days 30–35 Second FU: days 60–65	Negative culture rate: no difference at 30–35 days (*p* = 0.127); fluconazole group had a higher rate at 60–65 days (*p* = 0.016). Symptom improvement: Fluconazole group had lower rates of abnormal discharge, erythema (both follow-ups), and pruritus (second follow-up) (*p* < 0.05). No difference: burning, frequent urination, dysuria, and dyspareunia (*p* > 0.05) in both groups. Overall effectiveness: L. acidophilus was as effective as fluconazole in treating VVC symptoms but less effective in preventing recurrence.
Abbasi RCT Iran [36]	*n* = 47 treatment *n* = 22 control *n* = 25	FamiLact: 34.5 y/o Placebo group: 33.8 y/o	Treatment group: one capsule (500 mg) of FamiLact daily (1 × 10^9^ CFU) of *L. rhamnosus*, *L. casei*, *L. bulgaricus*, *L. acidophilus*, *B. breve*, *B. longum*, *S. Thermophilus*, and prebiotic Fructooligosaccharides). Control group: identical placebo capsule.	Sperm characteristics; sperm concentration; motility; normal morphology; DNA fragmentation.	80 days	Baseline: No significant differences in sperm characteristics between groups. FamiLact vs. control: FamiLact significantly improved sperm concentration (*p* = 0.01), motility (*p* = 0.04), and morphology (*p* = 0.03). Pre-/post-treatment (FamiLact): significant improvements in concentration (*p* = 0.004), motility (*p* = 0.003), morphology (*p* = 0.014), lipid peroxidation (*p* = 0.02), and DNA fragmentation (*p* = 0.005). Non-significant changes: semen volume and CMA3 positivity showed no difference. Placebo: only DNA fragmentation improved (*p* = 0.03); other parameters remained unchanged.
Li In vivo; in vitro China [29]	Human semen *n* = 18 Animal model: male: *n* = 18 female: *n* = 72	22–38 y/o Rats: 10 to 11 weeks old	Probiotics and bacterial strains treatment: *L. crispatus* Lcr-MH175; *Salmonella typhimurium* VNP20009; engineered *Salmonella typhimurium* VNP20009 DNase I; *Escherichia coli* O157:H7 (ATCC 12806).	In vitro: sperm motility Ca^2+^ concentration and adhesion to sperm. In vivo: pregnancy rates, uterine inflammatory factors (TLR-4/NF-κB, TNF-α, IL-1β), and apoptosis factors (Fas Ligand, Bax/Bcl-2).	2, 4, and 6 h	In vitro: *L. crispatus*, VNP20009, VNP20009 DNase I, and *E. coli* O157:H7 significantly inhibited sperm motility with negative impact on sperm intracellular Ca^2+^ concentration. In vivo: *: L. crispatus* and other tested bacteria significantly reduced pregnancy rates in rats. *L. crispatus* positively influenced maternal health and offspring development. Transplantation of *L. crispatus* maintained normal vaginal microbiota composition in healthy rats and markedly reduced the expression of uterine inflammatory factors (TLR-4/NF-κB, TNF-α, IL-1β) and apoptosis factors (Fas Ligand, Bax/Bcl-2) compared to other strains.
Ansari In vivo Iran [37]	96 male rats divided into eight groups	8-week-old rats	Control group: water and normal diet only. Sham group: compound solvent. Diabetic control group (DM): injected with STZ and gavaged with 1 mL of normal saline. Exp G-1 (Cynara): 1 mL of 400 mg/kg hydroalcoholic *Cynara scolymus* L. extract. Exp G-2 (BBL): 1 mL of *B. longum* (1 × 10^9^ CFU/mL/day). Exp G-3 (DM + Cynara): diabetic rats received 1 mL of 400 mg/kg hydroalcoholic *Cynara scolymus* L. extract by gavage. Exp G-4 (DM + BBL): diabetic rats received 1 mL of B. longum (1 × 10^9^ CFU/mL/day) by gavage. Exp G-5 (DM + Cynara + BBL): diabetic rats received 0.5 mL of 400 mg/kg hydroalcoholic *Cynara scolymus* L. extract and 0.5 mL of *B. longum* (1 × 10^9^ CFU/mL/day) by gavage.	Evaluation of biochemical parameters: FBS (calorimetric method), FSH, LH, and testosterone (ELISA); measurement of TAC (spectrophotometric method) and MDA (colorimetric method); sperm parameters: count, motility, viability (eosin–nigrosin staining), and morphology; gene expression analysis: RT-PCR for prm1, Bcl-2, and Caspase-9; stereological analysis: testis volume estimation (point counting), cell count (optical disector), and tubular length assessment.		Hydroalcoholic extract of *Cynara scolymus* L. combined with *B. longum* exhibited these beneficial effects compared to the diabetic group: Lowered levels: significant reduction in FBS (*p* < 0.001), MDA (*p* < 0.05), and Caspase-9 expression (1.33-fold). Increased serum markers: elevated TAC, LH, FSH, and testosterone levels (*p* < 0.05). Gene expression: upregulation of protamine (*p* = 0.016) and Bcl-2 (0.72-fold). Improved reproductive parameters: enhanced sexual lineage cell count, testis weight, sperm count, motility, normal morphology, and seminiferous tubule volume/length (*p* < 0.05).

Abbreviations: 16S rRNA seq = 16S ribosomal RNA sequencing; *B. adolescentis* = *Bifidobacterium adolescentis*; *B. bifidus* = *Bifidobacterium bifidum*; *B. breve* = *Bifidobacterium breve*; *B. longum* = *Bifidobacterium longum*; BICI = Body Image Concern Inventory; BMI = Body Mass Index; Exp = Experimental; FSFI = Female Sexual Function Index; FSH = follicle-stimulating hormone; FU = follow-up; G1, G2, G3, G4 = Group 1, Group 2, Group 3, Group 4; *L. acidophilus* = *Lactobacillus acidophilus*; *L. crispatus* = *Lactobacillus crispatus*; *L. fermentum* = *Lactobacillus fermentum*; *L. gasseri* = *Lactobacillus gasseri*; *L. plantarum* = *Lactobacillus plantarum*; *L. reuteri* = *Lactobacillus reuteri*; *L. rhamnosus* = *Lactobacillus rhamnosus*; LBG I, II, III = Lactobacillary Grade I, II, III; LDL-C = Low-Density Lipoprotein Cholesterol; LH = luteinizing hormone; RCT = randomized controlled trial; *S. thermophilus* = *Streptococcus thermophilus*; TAC = Total Antioxidant Capacity; TG = triglycerides.

## Data Availability

Not applicable.

## Supplementary Materials

The following supporting information can be downloaded at: https://www.mdpi.com/article/10.3390/biology14030286/s1, Supplement File S1: PRISMA 2020 checklist. Reference [75] is cited in the Supplementary Materials.

## Abbreviations

The following abbreviations are used in this manuscript:

FSFI	Female Sexual Function Index
BICI	Body Image Concern Inventory
TC	Total cholesterol
TG	Triglycerides
HDL	High-Density Lipoprotein
LDL	Low-Density Lipoprotein
GC-MS	Gas Chromatography–Mass Spectrometry
16S rRNA	16S ribosomal RNA
